# Tone disruptions in Mandarin post-stroke aphasia: an fNIRS study on Broca’s area using the auditory oddball paradigm

**DOI:** 10.1117/1.NPh.13.S1.S13006

**Published:** 2025-11-18

**Authors:** Likan Zhan, Wanfei Lv, Lijun Yin, Chenlu Guo, Chong Lu

**Affiliations:** a Beijing Language and Culture University, Cognitive Science and Allied Health School, Beijing, China; b Beijing Language and Culture University, Institute of Life and Health Sciences, Beijing, China; c Ministry of Education, Key Laboratory of Language and Cognitive Science, Beijing, China; d China Rehabilitation Research Center for Hearing and Speech Impairment, Beijing, China; e Tongji Hospital, Huazhong University of Science and Technology, Tongji Medical College, Wuhan, China; f Minzu University of China, College of International Education, Beijing, China

**Keywords:** post-stroke aphasia, auditory oddball paradigm; functional near-infrared spectroscopy, Broca’s area

## Abstract

**Significance:**

Tone processing is essential in tonal languages such as Mandarin. Understanding how post-stroke aphasia (PSA) affects tone perception can guide targeted rehabilitation, especially in linguistically unique populations.

**Aim:**

We aimed to explore the neural mechanisms of tone perception in Mandarin-speaking PSA patients and examine how brain network reorganization supports or hinders phonological processing.

**Approach:**

Using functional near-infrared spectroscopy (fNIRS) and a tone-based auditory oddball paradigm, we compared cortical activation and functional connectivity (FC) patterns between PSA patients and healthy controls.

**Results:**

Patients with PSA showed reduced/reversed hemodynamic responses in the left Broca’s area but increased FC with the right motor cortex. Despite this local hyperconnectivity, overall FC was lower in patients than in controls, especially among highly connected links (|r|>0.7), suggesting either compensatory or maladaptive reorganization. Moreover, deoxygenated hemoglobin changes in the left Broca’s area were positively associated with language function, as measured by aphasia quotient scores.

**Conclusions:**

The findings highlight altered auditory–motor network dynamics in PSA, with Broca’s area playing a central role in tone-based phonological processing. These results support the potential of fNIRS for clinical assessment and underscore the importance of accounting for network-level changes in aphasia rehabilitation strategies for tonal languages.

## 1 Introduction

Aphasia, a neurological language disorder resulting from brain damage, significantly impairs a person’s ability to comprehend or produce language. Post-stroke aphasia (PSA), in particular, is a common sign of stroke that is observed in about one-third of stroke patients and contributes to disease morbidity.^1^ This condition can manifest in various forms, but deficits in auditory comprehension are a hallmark of many aphasia subtypes, profoundly impacting patients’ ability to engage in meaningful communication.^2^ Individuals with PSA often experience difficulties in auditory attention and memory, which are critical cognitive functions for successful language comprehension.^3^ These difficulties not only hinder speech perception but also disrupt the ability to retain and process linguistic information over time, leading to severe communicative challenges. Among speakers of tonal languages, such as Mandarin Chinese, these difficulties are further undermined by the unique demands of tone perception. Mandarin utilizes pitch variations to convey lexical meaning, making tone discrimination a crucial aspect of language comprehension.^4^^,^^5^ Previous research has identified significant tone perception deficits in Chinese speakers with aphasia, particularly in distinguishing lexical tones that differ in pitch height and contour.^6^^–^^9^ These findings underscore the vulnerability of tonal processing mechanisms in PSA. Despite these insights, the neural mechanisms underlying tone perception deficits in aphasia remain poorly understood, particularly in tonal languages such as Mandarin. This gap in knowledge presents a critical barrier to developing targeted interventions for improving auditory comprehension in this population. Aphasia’s complexity stems from its intricate neural mechanisms, which involve both cortical and subcortical regions of the left hemisphere responsible for language processing.^10^ Tonal languages such as Mandarin introduce additional layers of complexity as pitch cues play a semantic role that is not as prominent in nontonal languages.^11^ Understanding how these mechanisms are disrupted in PSA is essential for advancing theoretical models of language processing and clinical practices. In this study, we aim to address these gaps by leveraging functional near-infrared spectroscopy (fNIRS) to investigate the neural underpinnings of lexical tone perception deficits in Mandarin-speaking individuals with PSA. By examining hemodynamic responses during tone processing, we aim to clarify how auditory attention and pitch perception interact in aphasia. This work enhances understanding of tone perception in aphasia and contributes to neurolinguistics by highlighting the specific demands of tonal language processing.

Aphasia is a language disorder that significantly impairs phonetic and phonological processing, particularly in phoneme selection, phonological rules, and speech articulation. These deficits often result from disruptions in speech planning and motor control, making communication challenging for individuals with the condition. For instance, studies have shown that phoneme errors are closely linked to impairments in speech–motor planning among aphasia patients, particularly in tasks involving phonological simplifications.^12^ In addition, the complexity of phonological processing plays a critical role in shaping therapeutic outcomes. Evidence suggests that treatments targeting more complex syllable structures yield superior results compared with interventions focused on simpler syllables.^13^ Among various subtypes of aphasia, nonfluent aphasia is particularly associated with deficits in phonological processing, which negatively affect tasks such as naming and narrative performance.^14^ These phonological challenges highlight the intricate interplay between sound-level processing and higher-level linguistic functions, emphasizing the need for integrative therapeutic strategies. To address these deficits, modern approaches to aphasia therapy are increasingly targeting phonological and semantic systems simultaneously. Such strategies have been shown to enhance naming accuracy and produce long-lasting therapeutic benefits, particularly for the verb retrieval task.^15^ Understanding the nature of phonological impairments in aphasia is thus essential not only for refining theoretical models of language processing but also for informing the development of effective, evidence-based interventions.

Mandarin Chinese is a prototypical tone language where the lexical meaning is conveyed through both segmental and tonal structures. Each syllable carries a tone, and differences in tone result in distinct meanings even for syllables that are segmentally identical. For instance, the syllable “/ma/” signifies “mother,” “hemp,” “horse,” and “scold” when produced with tone 1 (high-level), tone 2 (low-rising), tone 3 (falling-rising), and tone 4 (high-falling), respectively.^9^^,^^16^^,^^17^ The primary perceptual cue for distinguishing these tones is the fundamental frequency (f0) contour, which dominates over other auditory cues such as amplitude or vowel duration.^9^^,^^18^^,^^19^ Tones 2 and 3 present unique challenges for discrimination due to their overlapping pitch contours. Both tones involve rising or falling-rising pitch trajectories, making them phonetically similar, especially in connected speech or when produced with reduced articulatory clarity. This similarity leads to these two tones being the most perceptually confusable, both for native speakers and nonnative speakers.^20^ This inherent ambiguity makes tones 2 and 3 particularly informative materials for investigating tone perception deficits as they provide a robust test of an individual’s ability to resolve fine-grained pitch differences under challenging conditions. Behavioral studies confirm that tonal processing deficits are a core aspect of phonological impairment in Mandarin-speaking individuals with aphasia. These deficits affect both production and perception. For instance, even when tone productions are perceived as correct, acoustic analyses reveal underlying impairments such as flattened pitch contours and reduced slopes, indicating deficient motor programming of tonal targets.^9^ Similarly, perception studies have shown marked difficulties in distinguishing lexical tones, confirming a central deficit in processing tonal contrasts as linguistic units.^21^ Furthermore, broader auditory rhythm perception has been linked to speech fluency in aphasia,^22^ underscoring the tight coupling between auditory–phonological processing and expressive language abilities. These findings collectively demonstrate that tonal deficits are not peripheral but reflect a central vulnerability within the phonological system, affecting the encoding and retrieval of both segmental and suprasegmental features. These behavioral deficits are consistent with the central role of left-hemisphere language regions, including the superior temporal gyrus and Broca’s area, in processing lexical tones. Speech processing heavily depends on these regions, which support both perception and production of pitch variations carrying linguistic meaning. Together, these regions support the intricate functions of speech processing.^23^^–^^26^ Research into the neural mechanisms of tone processing reveals a complex interplay between acoustic and functional factors in the brain. Studies have indicated that the left hemisphere is primarily responsible for processing tonal information in linguistic contexts, whereas the right hemisphere, often linked to music processing, contributes to the perception of nonlinguistic acoustic features.^21^^,^^27^ In individuals with aphasia, pitch processing in speech can be significantly impaired. The extent of this impairment depends on the type and location of brain lesions. For instance, left hemisphere damage in fluent aphasics leads to notable deficits in tonal production and perception, as observed in languages such as Thai.^28^ Conversely, right hemisphere damage, such as in congenital amusia, results in general pitch-processing deficits, which can also affect tonal languages such as Mandarin.^29^ In addition, tonal deficits in aphasia can resemble impairments in consonant processing, with left hemisphere lesions affecting both tonal and consonantal features to similar extents.^30^ Although these studies have provided valuable insights, the specific neural mechanisms underlying fine tonal distinctions, such as those between Mandarin’s second and third tones, remain underexplored. Most existing research has focused on broader tone discrimination, leaving a gap in understanding the neural processes behind high-precision tonal contrasts critical to tonal languages.^31^ Further investigation is essential to better understand the cognitive and neural barriers faced by aphasic patients in processing these intricate tonal differences, paving the way for deeper insights into the challenges associated with tonal language contexts.

fNIRS is a noninvasive neuroimaging technique that measures brain activity by detecting changes in oxygenated hemoglobin (HbO), deoxygenated hemoglobin (DHb), and total hemoglobin(tHb) in cortical regions. Its portability, cost-effectiveness, and tolerance for movement artifacts make it particularly advantageous for studying populations such as PSA patients,^32^ children with autism,^33^ and individuals with schizophrenia,^34^ who may find traditional imaging methods, such as fMRI, challenging to tolerate.^35^^,^^36^ In language and speech research, fNIRS has demonstrated its utility in examining brain activity during tasks involving phonological, syntactic, and semantic processing.^37^^,^^38^ Furthermore, fNIRS has shown promise in studies of special populations, offering insights into their cognitive and linguistic impairments.^39^ In tonal languages such as Mandarin, fNIRS provides a valuable method for analyzing neural responses to pitch variations that differentiate lexical tones. Its ability to capture cortical responses to subtle phonetic contrasts, such as those between Mandarin’s second (rising tone) and third (dipping tone) tones, highlights its relevance for studying tone perception in both healthy individuals and patients with PSA. The current study aims to utilize fNIRS combined with an auditory oddball paradigm to investigate the neural mechanisms of tone processing, focusing on differences between aphasic patients and healthy controls. It is hypothesized that aphasic patients will exhibit reduced neural activity in language-related areas, such as the left Broca’s area, with attenuated changes in HbO, DHb, and tHb concentrations compared with healthy controls. By exploring these differences, the study seeks to contribute to a more nuanced understanding of the neural substrates of tonal processing in PSA.

## 2 Method

### 2.1 Subjects

A total of 14 healthy adults (10 males and 4 females) and 15 patients with PSA (10 males and 5 females) participated in this study. Healthy subjects were included based on the following criteria: aged 50 to 70, native Mandarin speakers with a minimum of elementary school education, and no history of vision or hearing impairment or significant neurological disorders. PSA patients were included if they were aged 50 to 70, had experienced a first-time stroke confirmed by CT or MRI, had stable vital signs, and demonstrated aphasia as diagnosed by experienced speech-language pathologists at Tongji Hospital’s Rehabilitation Medicine Department. Patients with co-occurring apraxia of speech or neurogenic speech disorders were excluded to ensure a clear focus on aphasia. The Chinese version of the Western Aphasia Battery (WAB) was employed to assess and classify aphasia, with all evaluations conducted by an experienced speech-language pathologist. This standardized assessment includes four linguistic subtests, which are critical for the diagnosis and classification of aphasia: (1) spontaneous speech: incorporating picture description to evaluate both content and fluency; (2) auditory comprehension: assessed through yes/no questions and execution of verbal commands; (3) repetition: involving the repetition of eight words and sentences of varying lengths; and (4) naming: tested through naming 20 objects and completing sentences with appropriate words. A total score below 93.8 out of 100 on the Chinese version of the WAB is considered indicative of aphasia.

Patients with significant comprehension deficits were excluded, ensuring that task demands would be manageable. Furthermore, cognitive function was evaluated using the Montreal Cognitive Assessment (MoCA), and those with moderate to severe cognitive impairments were excluded. The study was approved by the Institutional Ethical Committee of Beijing Language and Culture University (ID: 2024BYLL55). Ethical compliance was rigorously maintained, and all participants provided informed consent before participating. The control group was composed primarily of spouses or family members of aphasic patients, ensuring demographic comparability between groups. Detailed demographic information is presented in Table 1.

**Table 1:** Demographic information of participants.

Group	Age	Gender	Years of education	Handedness	Type of aphasia	Lesion	AQ
Aphasia group	69	M	13	Right	Broca	Parietal-occipital	39
Aphasia group	55	F	10	Right	Broca	Parietal-occipital	21.6
Aphasia group	54	M	12	Right	Anomic	Basal ganglia	80.6
Aphasia group	52	M	11	Right	Anomic	Basal ganglia	74
Aphasia group	53	M	9	Right	Anomic	Basal ganglia	82
Aphasia group	55	F	12	Right	Transcortical motor	Parietal	58
Aphasia group	56	M	9	Right	Anomic	Parietal	85
Aphasia group	64	M	9	Right	Anomic	Basal ganglia	78
Aphasia group	74	M	12	Right	Anomic	Parietal	86
Aphasia group	69	F	15	Right	Anomic	Basal ganglia	82
Aphasia group	57	F	16	Right	Anomic	Basal ganglia	83
Aphasia group	59	M	9	Right	Broca	Parietal	27.2
Aphasia group	48	M	10	Right	Anomic	Parietal-temporal	64.5
Aphasia group	52	F	12	Right	Anomic	Parietal-temporal	66.5
Aphasia group	56	M	15	Right	Anomic	Basal ganglia	81
Control group	55	M	9	Right	NA	NA	NA
Control group	61	M	12	Right	NA	NA	NA
Control group	52	M	10	Right	NA	NA	NA
Control group	55	F	9	Right	NA	NA	NA
Control group	54	M	12	Right	NA	NA	NA
Control group	71	M	15	Right	NA	NA	NA
Control group	64	F	16	Right	NA	NA	NA
Control group	50	M	12	Right	NA	NA	NA
Control group	67	M	10	Right	NA	NA	NA
Control group	54	F	9	Right	NA	NA	NA
Control group	67	F	9	Right	NA	NA	NA
Control group	56	M	12	Right	NA	NA	NA
Control group	57	M	15	Right	NA	NA	NA
Control group	56	M	12	Right	NA	NA	NA

M, male; F, female; AQ, aphasia quotient.

### 2.2 Procedure

**Fig. 1:**
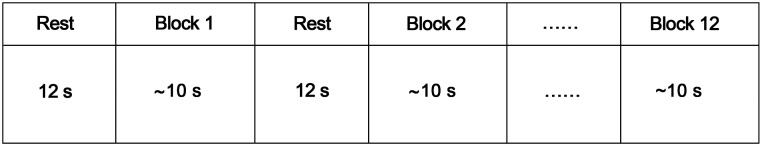
Auditory oddball paradigm procedure.

We utilized a passive auditory oddball task combined with fNIRS to investigate the neural responses to speech deviations. The analysis focused on HbO, DHb, and tHb. The stimuli consisted of a standard (nontarget) Mandarin phoneme /má/ (“ma”), with a frequency of 80%, and a deviant (target) Mandarin phoneme /mǎ/ (“ma”), with a frequency of 20%. The oddball paradigm employed in this study consisted of 120 auditory stimuli: 80 nontargets and 40 targets. The stimuli were delivered at an intensity of 80 dB HL using a pair of speakers placed 0.5 m away from the participant. Participants passively listened to the stimuli while mentally counting the target sounds. At the end of each experiment, participants were asked to report the number of target stimuli they had counted (see Fig. 1).

Each auditory stimulus had a duration of 500 ms, with inter-stimulus intervals varying randomly between 450 and 650 ms. The stimuli were presented in blocks, with 10 stimuli per block and an inter-block interval of 12 s. In total, 12 blocks were administered. The paradigm was implemented, and stimuli were presented using E-Prime 2.0 software^40^ (Psychology Software Tools, Pittsburgh, Pennsylvania, United States). Speech stimuli were recorded by a female native Mandarin speaker who had attained Grade 1-A proficiency in Standard Mandarin, as certified by the Putonghua Shuiping Ceshi (PSC) grading standards^41^ established by the Ministry of Education and the State Language Commission of China (1994). Recordings were made using Praat version 6.2.18 at a sampling rate of 44.1 kHz (16-bit, mono). This paradigm was adapted from the previous study.^42^ This design ensured a controlled delivery of stimuli to examine the neural mechanisms underlying speech processing.

### 2.3 fNIRS Recording

**Fig. 2:**
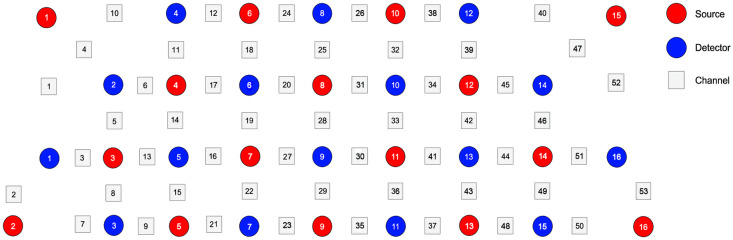
Optode arrangement and the entire device cover the prefrontal and temporal lobes of the brain.

We used a 53-channel fNIRS device (BS-7000, Wuhan Znion Technology Co., Ltd., Wuhan, China) to measure changes in HbO, DHb, and tHb concentrations during the oddball paradigm. The device features 16 emitter-detector pairs that operate at wavelengths of 760 and 850 nm with a sampling frequency of 10 Hz. The distance between each emitter and detector is ∼3  cm. The probes were positioned on the forehead according to the 10 to 20 system of electrode placement, commonly used in EEG and fNIRS studies.^43^ This system ensures accurate and standardized positioning of the probes relative to the underlying brain regions. Measurement channels are defined by the space between each emitter and detector pair, as shown in Fig. 2. For transparency, detailed MNI coordinates for each channel are provided in the Supplementary Material. Comprehensive details of the fNIRS configuration, including light source and detector placement, channel localization, and corresponding Brodmann areas, are provided in the Figs. S1–S2 and Table S1 in the Supplementary Material. This setup allows for precise data collection from key brain areas such as the dorsolateral prefrontal cortex and Broca’s area, which is in line with previous studies.^44^ For the current study, we focused on the left Broca’s area as the region of interest (ROI). The definition of this ROI is based on the channel configuration of the 53-channel fNIRS system, as detailed in Table 2. The left Broca’s area is critically involved in speech production and language processing. This ROI selection aligns with established methods in fNIRS research that target key language-related brain regions, particularly in relation to speech production and comprehension tasks. By analyzing the fNIRS data from these specific channels, we aim to explore the neural responses associated with tonal distinctions in Mandarin, a tonal language, in the left Broca’s area during the oddball task.

**Table 2:** Channel assignment for ROIs in the 53-channel fNIRS system.

Region of interest (ROI)	Channels
Left Broca’s area	2nd, 3rd, 5th, 7th, 8th, 13th channels
Right Broca’s area	44th, 49th, 50th, 46th, 51st, 53rd channels
Left dorsolateral prefrontal cortex (DLPFC)	6th, 11th, 14th, 17th, 18th, 20th channels
Right dorsolateral prefrontal cortex (DLPFC)	31st, 32nd, 34th, 39th, 42nd, 45th channels
Left frontal eye fields (FEF)	12th, 24th channels
Right frontal eye fields (FEF)	26th, 38th channels
Left frontopolar area (FP)	9th, 15th, 16th, 19th, 21st, 22nd, 23rd, 27th channels
Right frontopolar area (FP)	30th, 33rd, 35th, 36th, 37th, 41st, 43rd, 48th channels
Left motor and supplementary motor cortex	1st, 4th, 10th channels
Right motor and supplementary motor cortex	40th, 47th, 52nd channels

### 2.4 fNIRS Data Processing and Statistics

In the fNIRS data preprocessing, the data were analyzed using the Homer2 software package based on MATLAB.^44^ First, the raw intensity files were converted into the Homer2 (.nirs) format. Next, the raw fNIRS data were transformed into optical density (function: hmrIntensity2OD). Following the manufacturer’s recommendations, channels with a coefficient of variation >7.5% were considered bad channels and excluded from the analysis. Wavelet transform was applied to correct for motion artifacts (function: hmrMotion-CorrectWavelet) using the default quartile spacing of 0.1 as it was found to be the best option for motion correction. Motion artifacts were further removed using the motion artifact detection tool (function: hmrMotion-artifact), with the parameters set to tMotion = 3.0, tMask = 1.0, STDEVthresh = 20, and AMPthresh = 5.0. The signals were then band-pass filtered (function: hmrBandpassFilt) with a high-pass filter (HPF) of 0.010 Hz and a low-pass filter (LFP) of 0.10 Hz to remove baseline drift and physiological noise. Concentration changes in HbO and DHb were calculated based on the modified Beer–Lambert law. The hemodynamic responses were analyzed using the hmrBlockAvg function. A baseline correction was performed using a 2-s window immediately preceding each stimulus block. For statistical analysis, the mean concentration changes of HbO, DHb, and tHb for each channel were calculated across the 0 to 15 s time window following stimulus onset.

To examine the differences in HbO, DHb, and tHb concentrations between the aphasia group and the control group, a one-way analysis of variance (ANOVA) was conducted for each of these three measures within Broca’s area. The independent variable was the group (aphasia versus control), and the dependent variables were the concentration changes of HbO, DHb, and tHb. In addition, to explore the relationship between aphasia severity and neural activity, Pearson’s correlation analysis was conducted between the AQ and the HbO, DHb, and tHb concentration in Broca’s area for the aphasia group. A significance level of p<0.05 was adopted for all analyses.

Functional connectivity (FC)^45^ was assessed by examining the temporal correlations between hemodynamic signals from predefined ROIs.^46^ Ten ROIs were defined based on anatomical and functional landmarks: left and right Broca’s area, DLPFC, FEF, FP, and motor cortex. For each participant, the preprocessed time series of HbO, DHb, and tHb from all channels within an ROI were averaged to create a single representative time course for that ROI. This averaging process enhances the signal-to-noise ratio by integrating the activity across the functional region.^47^ For each hemoglobin species (HbO, DHb, and tHb), a 10×10 FC matrix was generated for each participant. Each element in this matrix represents the Pearson correlation coefficient (r) calculated between the averaged time courses of a pair of ROIs. To ensure normality for statistical comparisons, the Pearson correlation coefficients were converted to z-scores using Fisher’s z-transformation. Group-level statistical analysis focused on the FC between the left Broca’s area and each of the other ROIs. For each participant and each hemoglobin species (HbO, DHb, and tHb), Fisher’s z-transformed values were extracted for the connections between the left Broca’s area and the remaining nine ROIs. This resulted in nine sets of z-values per group for each connection (e.g., 15 z-values for the aphasia group and 14 z-values for the control group for the left Broca → right Broca connection). Each connection was then analyzed separately using a one-way ANOVA to compare FC strength between the aphasia and control groups. Statistical significance was set at p<0.05. Significant group differences may reflect either disruption of typical network communication due to the lesion or compensatory reorganization of brain networks.^48^

## 3 Results

### 3.1 Hemodynamic Response in the ROI

**Fig. 3:**
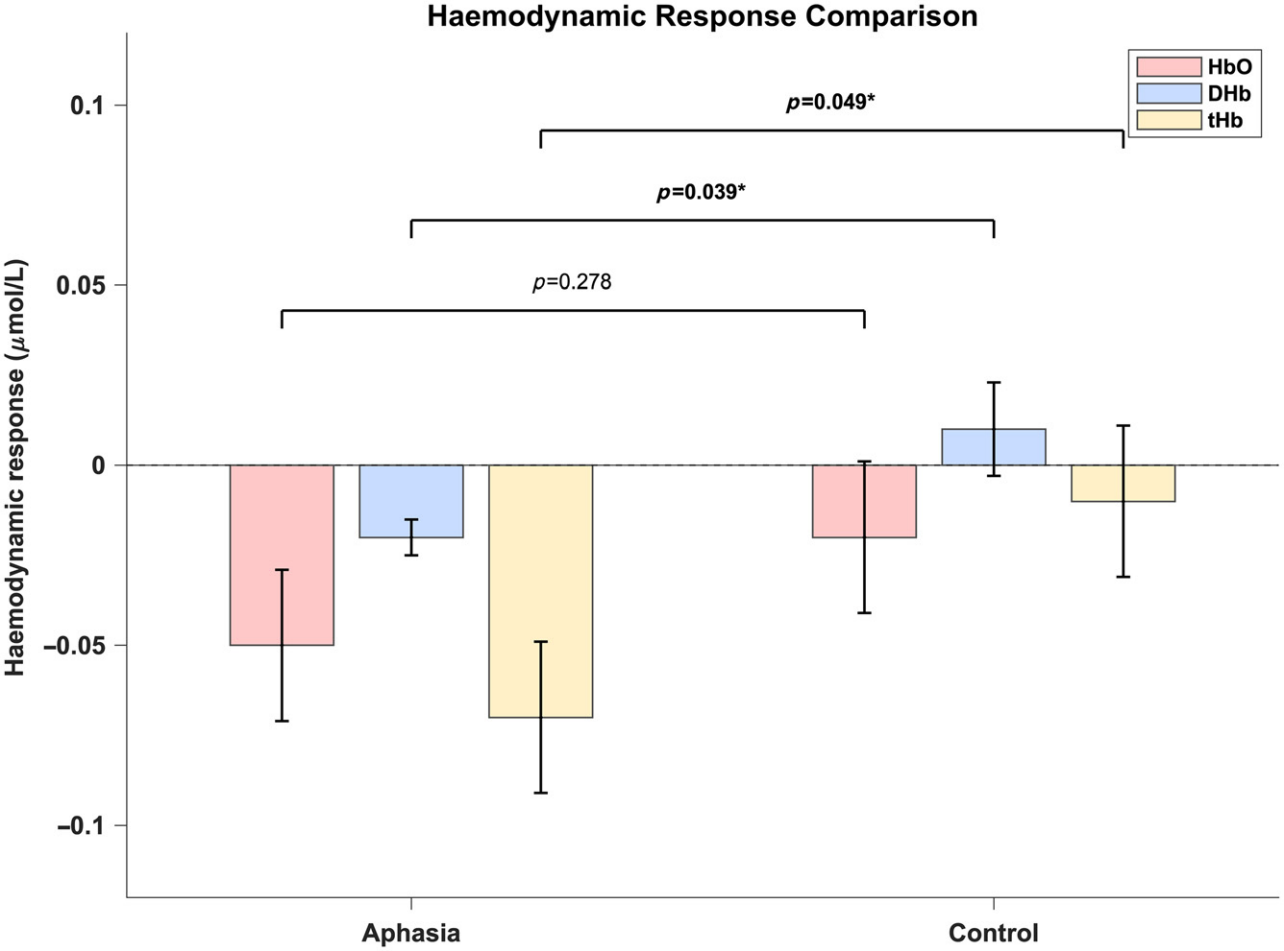
Comparison of HbO, DHb, and tHb concentrations between the aphasia and control groups. The y-axis represents concentration (μmol).

**Fig. 4:**
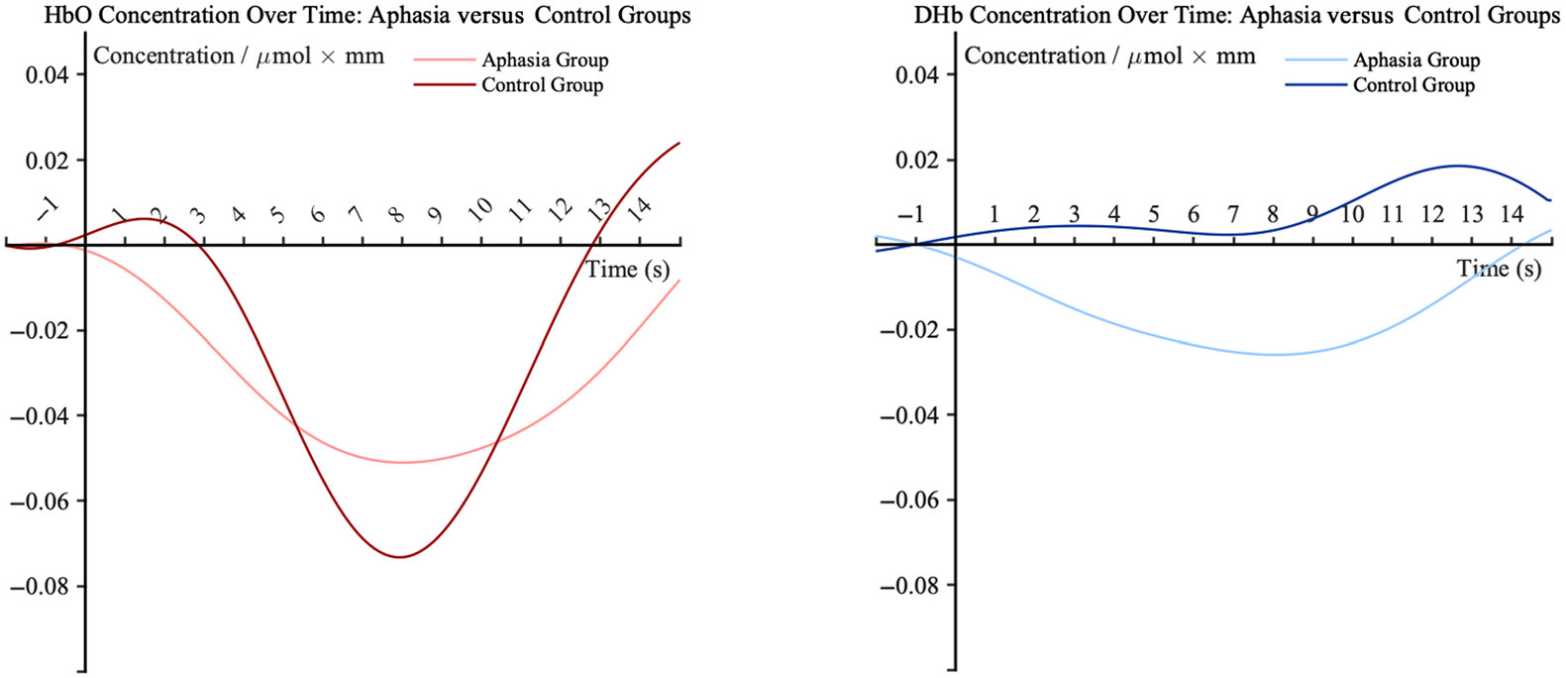
Time courses of hemoglobin concentration changes in the aphasia and control groups during the auditory oddball task at the left Broca’s area (average across subjects). The x-axis indicates time from 2 s before to 15 s after block onset; the y-axis indicates changes in hemoglobin concentration.

The ANOVA results indicated no significant difference in HbO concentrations between the aphasia group (M=−0.05, SE = 0.021) and the control group (M=−0.02, SE = 0.021), $F_{\left(1,27\right)}=1.228$, p=0.278, partial $\eta^{2}=0.043$, suggesting a small effect size. However, significant group differences were observed for DHb and tHb concentrations. Specifically, the aphasia group exhibited significantly lower DHb concentrations (M=−0.02, SE = 0.0052) compared with the control group (M=0.01, SE = 0.0134), $F_{\left(1,27\right)}=4.711$, p=0.039, partial $\eta^{2}=0.149$, indicating a medium effect size. Similarly, tHb concentrations were significantly reduced in the aphasia group (M=−0.07, SE = 0.0207) compared with the control group (M=−0.01, SE = 0.0214), $F_{\left(1,27\right)}=4.253$, p=0.049, partial $\eta^{2}=0.136$, also reflecting a medium effect size. These findings suggest significant differences in DHb and tHb concentrations between the two groups, whereas no such difference was observed for HbO concentrations. Figure 3 compares DHb and tHb concentrations between the aphasia and control groups. The bar charts illustrate the differences in hemoglobin concentrations between the two groups. The time courses for chromophore in Broca’s area are shown in Fig. 4.

**Fig. 5:**
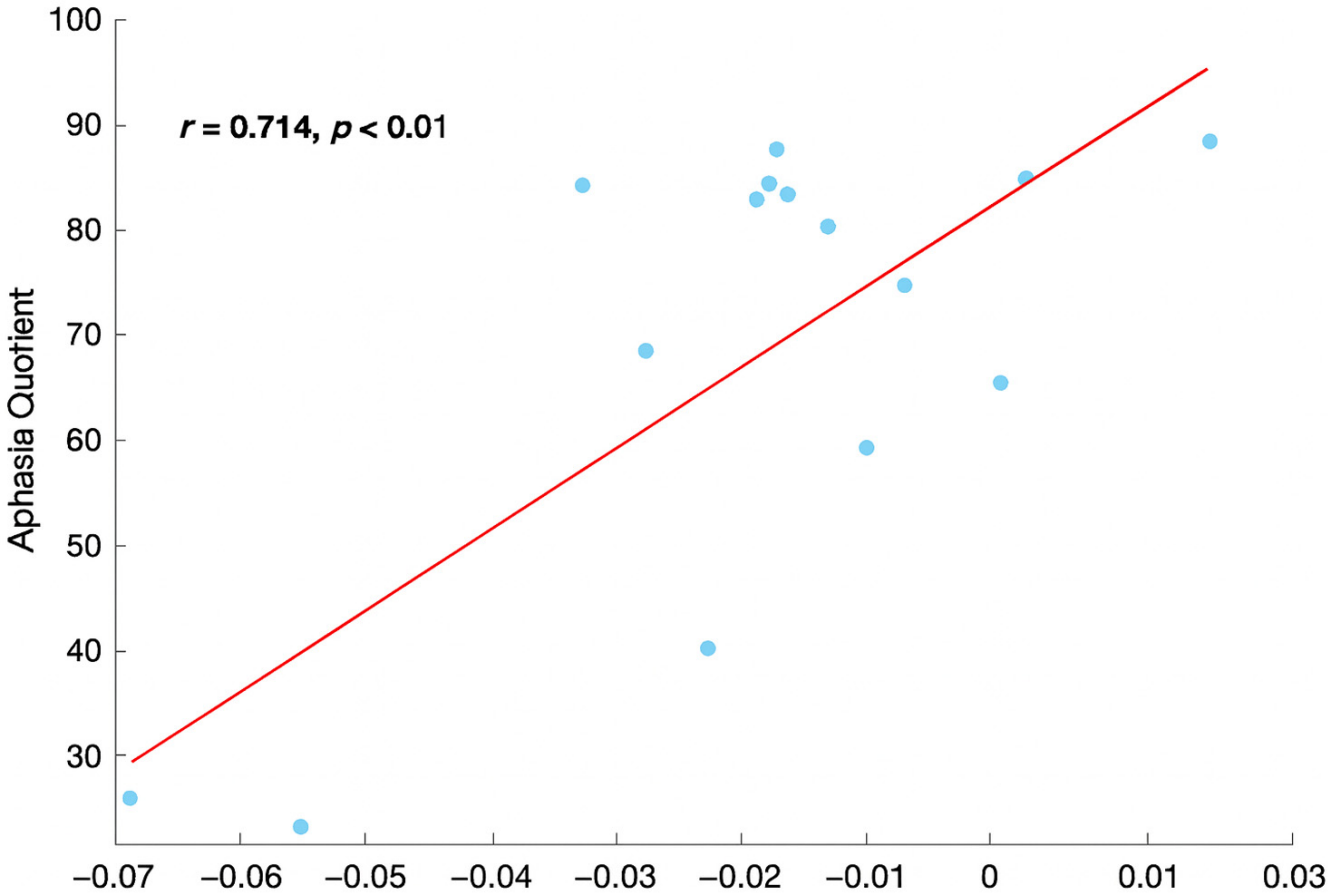
Correlation between DHb concentration and aphasia quotient.

The Pearson correlation analysis revealed a significant positive correlation between changes in DHb concentration and the AQ, with a Pearson correlation coefficient of r=0.714 (p<0.01). This relationship is further illustrated in Fig. 5. By contrast, no significant correlations were found for HbO or tHb (all p>0.05).

### 3.2 Functional Connectivity Analysis

Group comparisons of left Broca FC to other ROIs, based on HbO, DHb, and tHb (Fisher’s z-transformed r values), are summarized in Tables 3–5. For HbO-based FC, the aphasia group showed significantly stronger connectivity with right Broca and right Motor compared with controls. For DHb-based FC, connectivity with left FP was significantly higher in the control group. For tHb-based FC, the aphasia group exhibited significantly stronger connectivity with the right motor. No other ROI pairs showed significant group differences across the three hemodynamic measures. Detailed values are reported in Tables 3–5.

**Table 3:** Group comparisons of left Broca FC to other ROIs based on HbO (Fisher’s z-transformed r values).

ROI	Aphasia M ± SE	Control M ± SE	$F_{\left(1,27\right)}$	p	Partial $\eta^{2}$
Right Broca	1.28 ± 0.19	0.67 ± 0.11	7.23	0.012*	0.211
Left DLPFC	0.67 ± 0.20	0.45 ± 0.25	0.45	0.507	0.017
Right DLPFC	0.49 ± 0.14	0.08 ± 0.29	1.68	0.206	0.059
Left FEF	0.80 ± 0.29	0.59 ± 0.16	0.41	0.530	0.015
Right FEF	0.84 ± 0.31	0.56 ± 0.28	0.43	0.519	0.016
Left FP	0.96 ± 0.26	0.66 ± 0.26	0.67	0.421	0.024
Right FP	1.24 ± 0.20	0.76 ± 0.23	2.43	0.131	0.082
Left motor	0.53 ± 0.25	0.60 ± 0.22	0.04	0.845	0.001
Right motor	1.02 ± 0.17	0.31 ± 0.24	5.67	0.025*	0.174

* p<0.05.

**Table 4:** Group comparisons of left Broca FC to other ROIs based on DHb (Fisher’s z-transformed r values).

ROI	Aphasia M ± SE	Control M ± SE	$F_{\left(1,27\right)}$	p	Partial $\eta^{2}$
Right Broca	0.43 ± 0.17	0.87 ± 0.24	2.21	0.149	0.076
Left DLPFC	0.10 ± 0.30	0.59 ± 0.18	1.86	0.184	0.065
Right DLPFC	0.33 ± 0.25	0.46 ± 0.22	0.15	0.702	0.006
Left FEF	0.27 ± 0.17	0.25 ± 0.17	0.01	0.936	0.000
Right FEF	0.30 ± 0.28	−0.16 ± 0.19	1.76	0.196	0.061
Left FP	0.01 ± 0.21	0.63 ± 0.19	4.53	0.043*	0.144
Right FP	0.63 ± 0.21	0.38 ± 0.16	0.84	0.368	0.030
Left motor	0.36 ± 0.19	0.20 ± 0.17	0.39	0.540	0.014
Right motor	0.11 ± 0.20	0.22 ± 0.23	0.13	0.723	0.005

* p<0.05.

**Table 5:** Group comparisons of Left Broca FC to other ROIs based on tHb (Fisher’s z-transformed r values).

ROI	Aphasia M ± SE	Control M ± SE	$F_{\left(1,27\right)}$	p	Partial $\eta^{2}$
Right Broca	1.09 ± 0.20	0.87 ± 0.22	0.53	0.473	0.019
Left DLPFC	0.55 ± 0.20	0.98 ± 0.15	2.85	0.103	0.096
Right DLPFC	0.76 ± 0.26	0.05 ± 0.25	3.88	0.059	0.126
Left FEF	0.70 ± 0.27	0.54 ± 0.13	0.26	0.615	0.009
Right FEF	0.76 ± 0.32	0.44 ± 0.20	0.67	0.421	0.024
Left FP	0.84 ± 0.32	0.66 ± 0.24	0.19	0.669	0.007
Right FP	1.14 ± 0.23	0.87 ± 0.21	0.76	0.393	0.027
Left Motor	0.51 ± 0.25	0.66 ± 0.22	0.19	0.671	0.007
Right Motor	1.05 ± 0.15	0.27 ± 0.16	12.75	0.001**	0.321

** p<0.01.

**Fig. 6:**
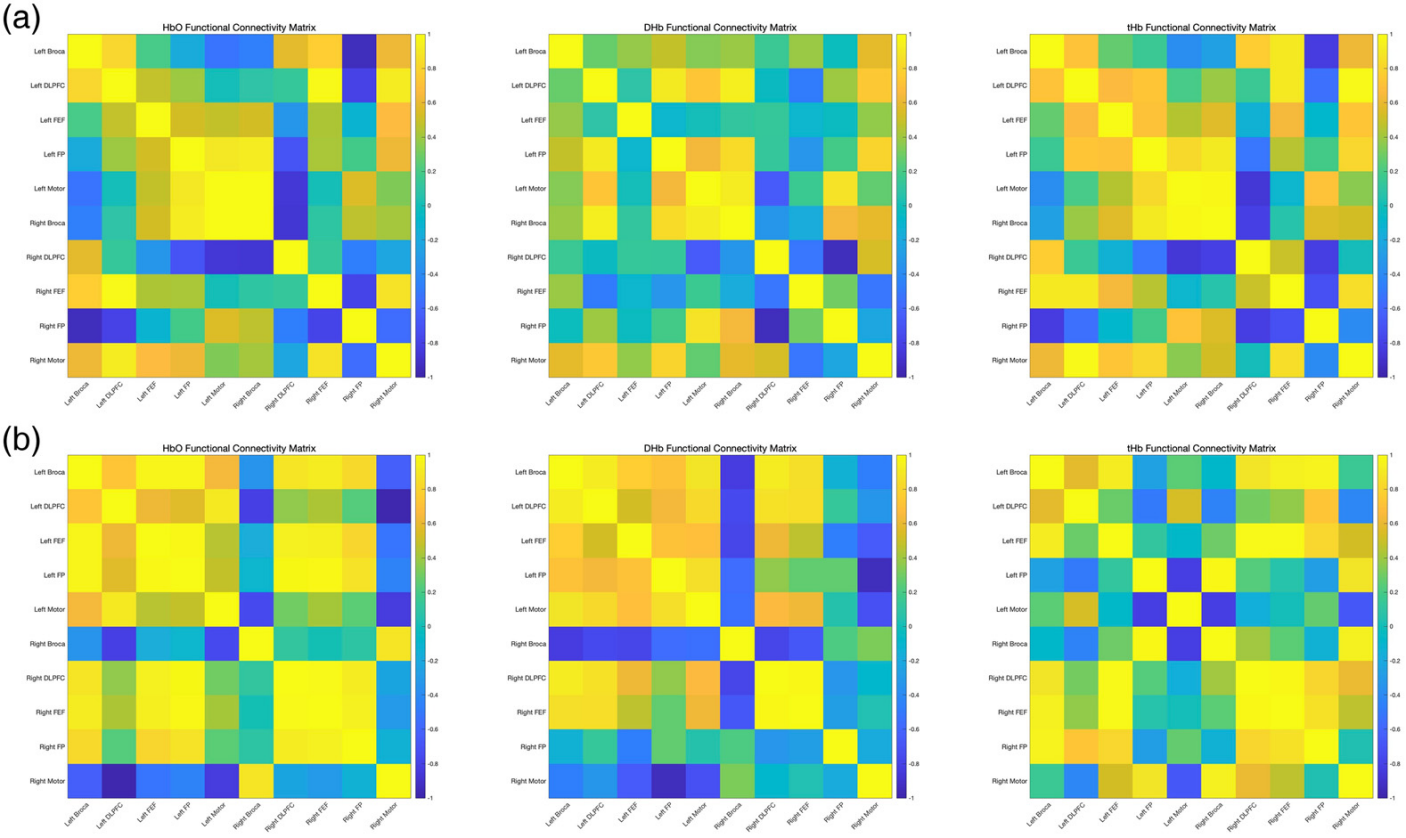
Group-level FC maps for the aphasia group (a) and control group (b) across three hemodynamic parameters: HbO, DHb, and tHb. The maps illustrate connectivity strength across all ROIs, derived from a 53-channel fNIRS system. Warmer colors indicate stronger positive correlations.

**Fig. 7:**
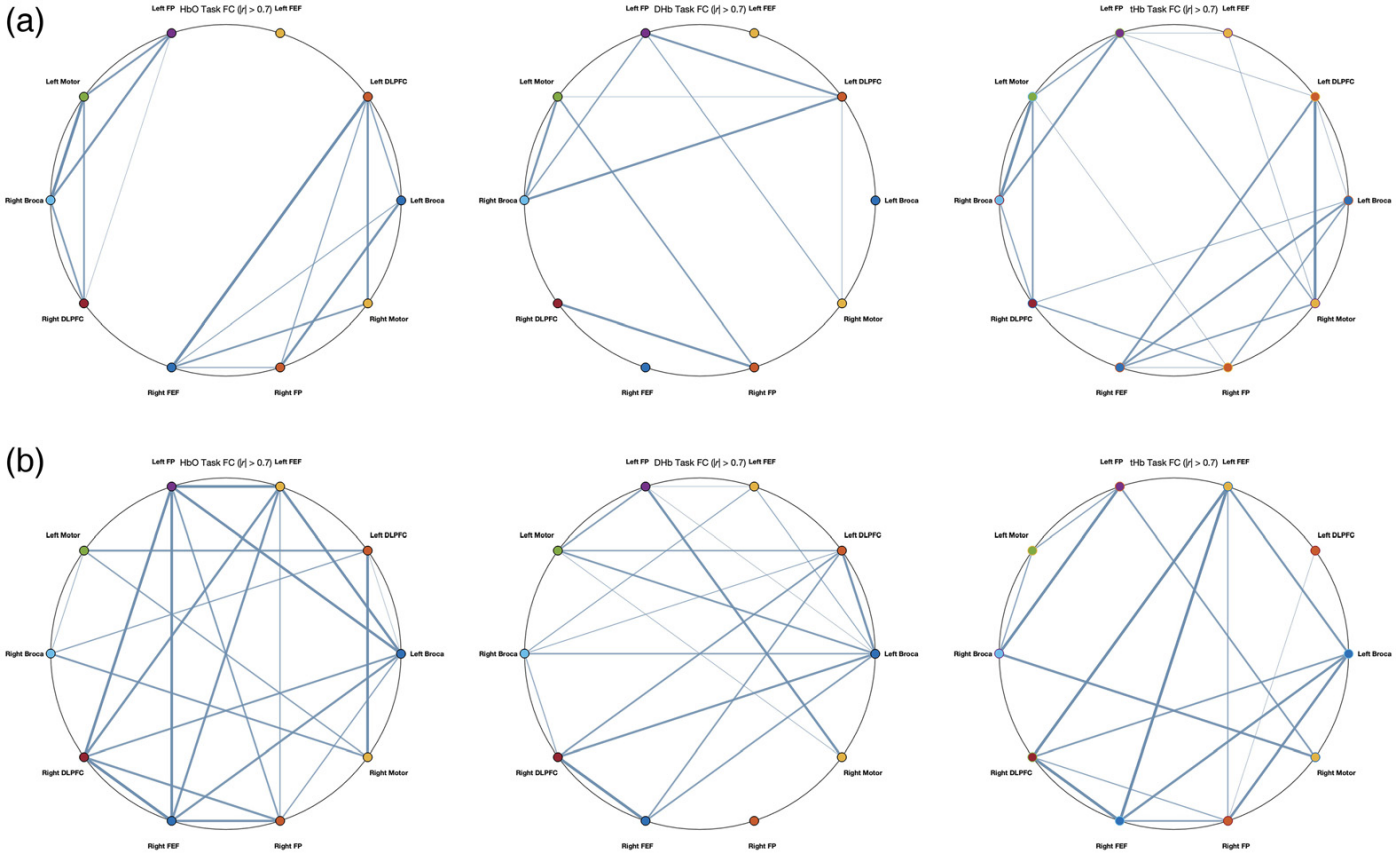
Group-level FC maps for the circular graph. Panel (a) displays data from individuals with aphasia, whereas panel (b) shows data from control participants. Connectivity is shown for three hemodynamic parameters: HbO, DHb, and tHb. Blue lines represent FC connections exceeding a threshold of |r|>0.7, whereas bold blue lines indicate stronger connections where |r|>0.9. The maps illustrate connectivity strength across all ROIs.

When examining individual connections between the left Broca’s area and the remaining ROIs, none of the connections reached statistical significance. Nevertheless, a consistent trend emerged: the aphasia group tended to show lower FC values than the control group, particularly in high-connectivity links (|r|>0.7). This trend was most apparent for connections involving the left Broca, right Broca, bilateral DLPFC, bilateral FEF, bilateral FP, and the left motor cortex. Together, these findings suggest that although global FC of the Left Broca’s area is significantly altered in aphasia, the reductions in individual connections did not reach significance, likely reflecting distributed but moderate changes across the network (see Figs. 6 and 7).

## 4 Discussion

In patients with PSA, we observed negative hemodynamic responses (decreased HbO and tHb) in Broca’s area during a tonal oddball task. Such “reverse activation” has been previously linked to impaired neurovascular coupling after stroke. For instance, Lu et al.^49^ found reduced HbO in Broca’s area of aphasic patients during a Stroop task, indicating compromised hemodynamic reactivity. Similarly, Li et al.^50^ reported diminished or negative HbO responses in global aphasia, correlating with poorer naming performance. Beyond stroke, atypical negative responses have been observed under strong auditory stimulation^51^ and may reflect disrupted vascular regulation, as also suggested in studies of immature neurovascular systems.^52^ Collectively, these findings indicate that the negative responses in our cohort likely reflect pathological neurovascular coupling in Broca’s area, potentially due to both reduced metabolic demand and impaired vascular function. Our study reveals distinct patterns of network reorganization in PSA, characterized by hemodynamic-specific alterations in FC originating from left Broca’s area. Patients exhibited significantly stronger HbO-based connectivity with right Broca and right motor regions, alongside enhanced tHb connectivity specifically with right motor cortex. This pattern suggests compensatory recruitment of contralateral homologues and motor integration pathways, consistent with interhemispheric compensation models.^53^ Conversely, controls demonstrated stronger DHb-based connectivity with left frontoparietal regions, indicating potentially disrupted metabolic coordination within ipsilateral cognitive control networks in patients.^54^ The correlation between reduced DHb changes and poorer aphasia quotient scores reinforces the clinical relevance of DHb measures, suggesting impaired neurovascular coupling underlies language deficits.^55^ The divergent patterns across hemodynamic measures highlight the importance of multiparametric assessment in capturing the complexity of post-stroke reorganization. The observed connectivity patterns reflect both adaptive reorganization through contralateral recruitment and potential maladaptation in ipsilateral networks, offering a refined framework for understanding neural dynamics in PSA recovery.

The reduced DHb and tHb responses observed in the left Broca’s area of Mandarin-speaking PSA patients align with established evidence linking stroke-induced metabolic dysfunction to impaired neural processing. These hemodynamic abnormalities suggest compromised neurovascular coupling, which may underlie the tonal perception deficits widely documented in behavioral studies of PSA speakers.^6^^,^^9^^,^^20^ A consistent trend emerged in FC: the aphasia group showed generally lower FC values than controls, particularly in high-connectivity links (|r|>0.7), suggesting broad network disruption following stroke. Nonetheless, our findings also converge with neuroimaging research emphasizing the role of left-hemisphere regions—such as the left inferior frontal sulcus and superior temporal areas—in categorical and fine-grained acoustic processing of tones.^28^ Importantly, even in patients without direct lesions in Broca’s area, aberrant responses were detected, implying system-level dysfunction. This supports the view that Broca’s area contributes not only to speech production but also to high-level perceptual and attentional integration of pitch-based phonological information.^56^^–^^58^ Although local activation was diminished, increased FC with the right motor cortex suggests selective adaptive reorganization, consistent with reports of contralesional recruitment after left-hemisphere damage.^59^^–^^63^ This dissociation between widespread FC reduction and specific hyperconnectivity reflects a complex pattern of plasticity that merits further investigation in tonal language contexts.

The correlation between DHb changes and language performance (AQ) highlights Broca’s area as a critical node for functional recovery, suggesting that modulating its activity may improve tonal processing outcomes in PSA patients. Noninvasive brain stimulation techniques, such as transcranial magnetic stimulation (TMS) or transcranial direct current stimulation (tDCS), offer promising avenues for enhancing excitability within this region and facilitating neural plasticity.^64^^–^^67^ Future studies could apply excitatory stimulation over Broca’s area—or inhibitory stimulation over contralateral homologues—to rebalance interhemispheric interactions and improve auditory attention and tone discrimination. The increased connectivity with the right motor cortex further suggests that network-level modulation might yield benefits beyond local stimulation. By integrating neuromodulation with tone-based rehabilitation paradigms, researchers can assess whether enhancing Broca’s area functionality promotes recovery of pitch contour processing and phonological encoding. Such approaches would not only advance theoretical understanding of tonal processing networks but also contribute to developing targeted interventions for Mandarin-speaking aphasia patients with tone perception deficits.

This study has several limitations. First, the relatively small sample size may affect the generalizability of the findings, underscoring the need for validation with larger cohorts. Second, the sole use of fNIRS has limited temporal resolution for capturing rapid neural oscillations; complementing it with EEG in future work could better resolve spatiotemporal dynamics. Third, the lack of correlation between AQ scores and HbO/tHb—in contrast to the significant association with DHb—may reflect post-stroke neurovascular uncoupling, suggesting DHb as a potentially more robust metabolic biomarker in chronic aphasia. Furthermore, variations among aphasia subtypes were not accounted for, which may underlie distinct neural patterns and recovery trajectories. Future research should prioritize larger and more diverse participant samples and incorporate multimodal neuroimaging (e.g., fNIRS-EEG) to simultaneously capture spatial and temporal aspects of neural processing. The application of GLM-based analyses should also be explored to enhance inference of brain–behavior relationships. Further investigation into tonal and phonetic contrasts across different aphasia subtypes may inform more individualized rehabilitation strategies. Clinically, these findings support the integration of tone-based tasks into assessment and intervention frameworks for Mandarin-speaking aphasia patients. Clinicians and speech-language pathologists should consider incorporating tone perception tasks into adapted versions of tools such as the WAB to better capture the specific linguistic deficits in this population. Furthermore, therapies targeting auditory attention could facilitate functional reorganization within language networks. Such a holistic and linguistically tailored approach aligns closely with the neurocognitive demands of tonal processing and may contribute to improved functional outcomes in language recovery.

## 5 Conclusion

This study provides evidence that PSA impairs tonal language processing in Mandarin speakers, particularly in the left Broca’s area. We observed reduced DHb and tHb responses in this region, indicating impaired neurovascular function during pitch-based phonological processing. Despite reduced local activation, increased FC between left Broca’s area and the right motor cortex suggests compensatory interhemispheric reorganization after stroke. The correlation between DHb changes and AQ scores further links these neural responses to language impairment severity. Using fNIRS with a tonal oddball paradigm, this work offers a promising approach to probe neural mechanisms of tonal processing. These findings support Broca’s area’s role in tonal perception and highlight the potential for neuromodulatory interventions targeting this region to improve tone processing recovery. Future studies could explore noninvasive brain stimulation, such as TMS or tDCS, over Broca’s area to directly modulate its activity and enhance tonal language rehabilitation in Mandarin-speaking PSA patients.

## Supplementary Material

10.1117/1.NPh.13.S1.S13006.s01

## Data Availability

The datasets generated and analyzed during the current study are available from the corresponding author upon reasonable request. The code used for analysis was written in MATLAB and can be obtained by contacting the corresponding author.

## References

[1] Yang Z.-H. et al., “Neuroanatomic correlation of the post-stroke aphasias studied with imaging,” Neurol. Res. 30(4), 356–360 (2008). 10.1179/174313208X300332 18544251

[2] Caplan D. Marshall J. C., Language: Structure, Processing, and Disorders, The MIT Press (1992).

[3] Marshall C. R. et al., “Primary progressive aphasia: a clinical approach,” J. Neurol. 265(6), 1474–1490 (2018). 10.1007/s00415-018-8762-6 29392464 PMC5990560

[4] Klein D. et al., “A cross-linguistic PET study of tone perception in Mandarin Chinese and English speakers,” NeuroImage 13(4), 646–653 (2001). NEIMEF 1053-8119 10.1006/nimg.2000.0738 11305893

[5] Francis A. L. et al., “Perceptual learning of Cantonese lexical tones by tone and non-tone language speakers,” J. Phon. 36(2), 268–294 (2008). JPHNB9 10.1016/j.wocn.2007.06.005

[6] Eng N. et al., “Tone perception deficits in Chinese-speaking Broca’s aphasics,” Aphasiology 10(6), 649–656 (1996). 10.1080/02687039608248441

[r7] Bl T. et al., “Tonal and orthographic analysis in a Cantonese-speaking individual with nonfluent/agrammatic variant primary progressive aphasia,” Neurocase 28(1), 1–10 (2022). 10.1080/13554794.2021.1925302 34404317 PMC9345301

[r8] Zhang W. Liao Y. Chang H., “Categorical perception of lexical tones in Chinese people with post-stroke aphasia,” Clin. Linguist. Phon. 37(12), 1069–1090 (2023). 10.1080/02699206.2022.2138785 36373592

[9] Chen W. et al., “Tone and vowel disruptions in Mandarin aphasia and apraxia of speech,” Clin. Linguist. Phon. 37(8), 742–765 (2023). 10.1080/02699206.2022.2081611 35656744

[10] van Son D. et al., “Frontal EEG theta/beta ratio during mind wandering episodes,” Biol. Psychol. 140, 19–27 (2019). 10.1016/j.biopsycho.2018.11.003 30458199

[11] Hallé P. A. Chang Y.-C. Best C. T., “Identification and discrimination of Mandarin Chinese tones by Mandarin Chinese vs. French listeners,” J. Phon. 32(3), 395–421 (2004). JPHNB9 10.1016/S0095-4470(03)00016-0

[12] Galluzzi C. et al., “Phonological simplifications, apraxia of speech and the interaction between phonological and phonetic processing,” Neuropsychologia 71, 64–83 (2015). NUPSA6 0028-3932 10.1016/j.neuropsychologia.2015.03.007 25772602

[13] Maas E. et al., “Treatment of sound errors in aphasia and apraxia of speech: effects of phonological complexity,” Aphasiology 16(4–6), 609–622 (2002). 10.1080/02687030244000266 22787286 PMC3392129

[14] Gordon J. K., “Measuring the lexical semantics of picture description in aphasia,” Aphasiology 22(7-8), 839–852 (2008). 10.1080/02687030701820063 22399832 PMC3293396

[15] Park E. J. et al., “Association between phonation and the vowel quadrilateral in patients with stroke: a retrospective observational study,” Medicine 99(39), e22236 (2020). MEDIAV 0025-7974 10.1097/MD.0000000000022236 32991418 PMC7523773

[16] Duanmu S., The Phonology of Standard Chinese, Oxford University Press (2002).

[17] Francis A. L. Ciocca V. Chit Ng B. K., “On the (non)categorical perception of lexical tones,” Percept. Psychophys. 65(7), 1029–1044 (2003). PEPSBJ 0031-5117 10.3758/BF03194832 14674631

[18] Fu Q.-J. Zeng F.-G., “Identification of temporal envelope cues in Chinese tone recognition,” Asia Pac. J. Speech Lang. Hear. 5(1), 45–57 (2000). 10.1179/136132800807547582

[19] Whalen D. H. Xu Y., “Information for Mandarin tones in the amplitude contour and in brief segments,” Phonetica 49(1), 25–47 (1992). PHNTAW 0031-8388 10.1159/000261901 1603839

[20] Shen X. S. Lin M., “A perceptual study of Mandarin tones 2 and 3,” Lang. Speech 34(2), 145–156 (1991). LGSHA4 10.1177/002383099103400202

[21] Li Q. et al., “Lexical tone perception in Mandarin Chinese speakers with aphasia,” Chinese J. Appl. Linguist. 44(1), 54–67 (2021). 10.1515/CJAL-2021-0004

[22] Stefaniak J. D. et al., “Auditory beat perception is related to speech output fluency in post-stroke aphasia,” Sci. Rep. 11(1), 3168 (2021). SRCEC3 2045-2322 10.1038/s41598-021-82809-w 33542379 PMC7862238

[23] Obleser J. et al., “Segregation of vowels and consonants in human auditory cortex: evidence for distributed hierarchical organization,” Front. Psychol. 1, 232 (2010).1664-1078 10.3389/fpsyg.2010.00232 21738513 PMC3125530

[r24] Bouchard K. E. et al., “Functional organization of human sensorimotor cortex for speech articulation,” Nature 495(7441), 327–332 (2013). 10.1038/nature11911 23426266 PMC3606666

[r25] Chang E. F. et al., “Categorical speech representation in human superior temporal gyrus,” Nat. Neurosci. 13(11), 1428–1432 (2010). NANEFN 1097-6256 10.1038/nn.2641 20890293 PMC2967728

[26] Formisano E. et al., “‘Who’ is saying ‘what’? Brain-based decoding of human voice and speech,” Science 322(5903), 970–973 (2008). SCIEAS 0036-8075 10.1126/science.1164318 18988858

[27] Myers E. B. et al., “Inferior frontal regions underlie the perception of phonetic category invariance,” Psychol. Sci. 20(7), 895–903 (2009).1467-9280 10.1111/j.1467-9280.2009.02380.x 19515116 PMC2851201

[28] Gandour J. et al., “Lexical tones in Thai after unilateral brain damage,” Brain Lang. 43(2), 275–307 (1992). 10.1016/0093-934X(92)90131-W 1393523

[29] Tillmann B. et al., “Congenital amusia (or tone-deafness) interferes with pitch processing in tone languages,” Front. Psychol. 2, 120 (2011).1664-1078 10.3389/fpsyg.2011.00120 21734894 PMC3119887

[30] Packard J. L., “Tone production deficits in nonfluent aphasic Chinese speech,” Brain Lang. 29(2), 212–223 (1986). 10.1016/0093-934X(86)90045-3 3790980

[31] Blumstein S. E. et al., “The perception and production of Voice-Onset Time in aphasia,” Neuropsychologia 15(3), 371–383 (1977). NUPSA6 0028-3932 10.1016/0028-3932(77)90089-6 854156

[32] Meier E. L. et al., “Resting-state connectivity in acute and subacute poststroke aphasia: a functional near-infrared spectroscopy pilot study,” Brain Connect. 13(8), 441–452 (2023). 10.1089/brain.2022.0065 37097208 PMC10618818

[33] Han Y. M. Y. et al., “Neurophysiological and behavioral effects of multisession prefrontal tDCS and concurrent cognitive remediation training in patients with autism spectrum disorder (ASD): a double-blind, randomized controlled fNIRS study,” Brain Stimul. 15(2), 414–425 (2022). 10.1016/j.brs.2022.02.004 35181532

[34] Kumar V. et al., “Functional near infra-red spectroscopy (fNIRS) in schizophrenia: a review,” Asian J. Psychiatr. 27, 18–31 (2017). 10.1016/j.ajp.2017.02.009 28558892

[35] Ferrari M. Quaresima V., “A brief review on the history of human functional near-infrared spectroscopy (fNIRS) development and fields of application,” NeuroImage 63(2), 921–935 (2012). NEIMEF 1053-8119 10.1016/j.neuroimage.2012.03.049 22510258

[36] Pinti P. et al., “The present and future use of functional near-infrared spectroscopy (fNIRS) for cognitive neuroscience,” Ann. N. Y. Acad. Sci. 1464(1), 5–29 (2020). ANYAA9 0077-8923 10.1111/nyas.13948 30085354 PMC6367070

[37] Sun X. et al., “Morphological and phonological processing in English monolingual, Chinese-English bilingual, and Spanish-English bilingual children: an fNIRS neuroimaging dataset,” Data Brief 42, 108048 (2022). 10.1016/j.dib.2022.108048 35313503 PMC8933821

[38] Zhao L. et al., “Syntactic and semantic processing in Japanese sentence reading: a research using functional near-infrared spectroscopy (fNIRS),” J. Psycholinguist. Res. 52(1), 57–73 (2023). JPLRB7 0090-6905 10.1007/s10936-021-09818-8 34775544

[39] Ali J. et al., “Near-Infrared Spectroscopy (NIRS) for cerebral and tissue oximetry: analysis of evolving applications,” J. Cardiothorac. Vasc. Anesth. 36(8 Pt A), 2758–2766 (2022). JCVAEK 1053-0770 10.1053/j.jvca.2021.07.015 34362641

[40] “E-Prime^®^ Stimulus Presentation Software | Psychology Software Tools,” Psychology Software Tools, Inc., https://pstnet.com/products/e-prime/ (accessed 16 September 2025).

[41] PSC proficiency test, KLC International Institute Pte Ltd, https://klc.edu.sg/psc-proficiency-test/.

[42] Yaman H. et al., “fNIRS-based evaluation of the impact of SARS-CoV-2 infection central auditory processing,” Brain Behav. 13(12), e3303 (2023). 10.1002/brb3.3303 37908040 PMC10726898

[43] Okamoto M. et al., “Three-dimensional probabilistic anatomical cranio-cerebral correlation via the international 10–20 system oriented for transcranial functional brain mapping,” NeuroImage 21(1), 99–111 (2004). NEIMEF 1053-8119 10.1016/j.neuroimage.2003.08.026 14741647

[44] Zhao Q. et al., “Anxiety symptoms without depression are associated with cognitive control network (CNN) dysfunction: an fNIRS study,” Psychophysiology 61(7), e14564 (2024). PSPHAF 0048-5772 10.1111/psyp.14564 38487932

[45] Friston K. J., “Functional and effective connectivity: a review,” Brain Connect. 1(1), 13–36 (2011). 10.1089/brain.2011.0008 22432952

[46] Yücel M. A. et al., “Best practices for fNIRS publications,” Neurophotonics 8(1), 012101 (2021). 10.1117/1.NPh.8.1.012101 33442557 PMC7793571

[47] Pinti P. et al., “A review on the use of wearable functional near-infrared spectroscopy in naturalistic environments,” Jpn. Psychol. Res. 60(4), 347–373 (2018). 10.1111/jpr.12206 30643322 PMC6329605

[48] Baldassarre A. et al., “Large-scale changes in network interactions as a physiological signature of spatial neglect,” Brain 137(12), 3267–3283 (2014). BRAIAK 0006-8950 10.1093/brain/awu297 25367028 PMC4240302

[49] Lu C. et al., “Unveiling cognitive interference: fNIRS insights into poststroke aphasia during stroop tasks,” Neural Plast. 2025, 1456201 (2025). 10.1155/np/1456201 40201621 PMC11976049

[50] Li H. et al., “Language reorganization patterns in global aphasia-evidence from fNIRS,” Front. Neurol. 13, 1025384 (2022). 10.3389/fneur.2022.1025384 36686505 PMC9853054

[51] Muñoz V. et al., “Neurovascular coupling during auditory stimulation: event-related potentials and fNIRS hemodynamic,” Brain Struct. Funct. 228(8), 1943–1961 (2023). 10.1007/s00429-023-02698-9 37658858 PMC10517045

[52] de Roever I. et al., “Investigation of the pattern of the hemodynamic response as measured by functional near-infrared spectroscopy (fNIRS) studies in newborns, less than a month old: a systematic review,” Front. Hum. Neurosci. 13, 371 (2018). 10.3389/fnhum.2018.00371 PMC6176492 30333736

[53] Dimyan M. A. Cohen L. G., “Neuroplasticity in the context of motor rehabilitation after stroke,” Nat. Rev. Neurol. 7(2), 76–85 (2011). 10.1038/nrneurol.2010.200 21243015 PMC4886719

[54] Heiss W.-D. Thiel A., “A proposed regional hierarchy in recovery of post-stroke aphasia,” Brain Lang. 98(1), 118–123 (2006). 10.1016/j.bandl.2006.02.002 16564566

[55] Das Y. et al., “Wavelet-based neurovascular coupling can predict brain abnormalities in neonatal encephalopathy,” NeuroImage: Clin. 32, 102856 (2021). 10.1016/j.nicl.2021.102856 34715603 PMC8564674

[56] Gandour J. et al., “A cross-linguistic FMRI study of spectral and temporal cues underlying phonological processing,” J. Cogn. Neurosci. 14(7), 1076–1087 (2002). JCONEO 0898-929X 10.1162/089892902320474526 12419130

[r57] Hickok G. Poeppel D., “The cortical organization of speech processing,” Nat. Rev. Neurosci. 8(5), 393–402 (2007). NRNAAN 1471-003X 10.1038/nrn2113 17431404

[58] Wang Y. et al., “fMRI evidence for cortical modification during learning of Mandarin lexical tone,” J. Cogn. Neurosci. 15(7), 1019–1027 (2003). JCONEO 0898-929X 10.1162/089892903770007407 14614812

[59] Klingbeil J. et al., “EP 6. Longitudinal resting state functional connectivity patterns in the early phase of recovery from Aphasia in temporoparietal stroke,” Clin. Neurophysiol. 127, e235 (2016). CNEUFU 1388-2457 10.1016/j.clinph.2016.05.062

[r60] Fiori V. et al., “P103 Functional connectivity changes after bilateral tDCS over the frontal region: preliminary data from aphasia,” Clin. Neurophysiol. 128, e62 (2017). CNEUFU 1388-2457 10.1016/j.clinph.2016.10.227

[r61] Christman S. Boutsen F., Recovery of Language after Stroke or Trauma in Adults, pp. 401–414, Elsevier (2006).

[r62] Mylius V. et al., “Stroke rehabilitation using noninvasive cortical stimulation: aphasia,” Expert Rev. Neurotherap. 13, 973–982 (2012). 10.1586/ern.12.76 23002940

[63] Mohr B., “Neuroplasticity and functional recovery after intensive language therapy in chronic post stroke aphasia: which factors are relevant?” Front. Hum. Neurosci. 11, 332 (2017). 10.3389/fnhum.2017.00332 28701937 PMC5487528

[64] Fridriksson J. Hillis A. E., “Current approaches to the treatment of post-stroke aphasia,” J. Stroke 23(2), 183–201 (2021). 10.5853/jos.2020.05015 34102754 PMC8189855

[r65] Naeser M. A. et al., “Improved picture naming in chronic aphasia after TMS to part of right Broca’s area: an open-protocol study,” Brain Lang. 93(1), 95–105 (2005). 10.1016/j.bandl.2004.08.004 15766771

[r66] Wang C. et al., “The therapeutic effect of transcranial magnetic stimulation on post-stroke aphasia and the optimal treatment parameters: a meta-analysis,” Arch. Phys. Med. Rehabil. 105(7), 1388–1398 (2024). APMHAI 0003-9993 10.1016/j.apmr.2023.11.006 37984539

[67] Ilkhani M. et al., “The effect of low-frequency repetitive transcranial magnetic stimulation (rTMS) on the treatment of aphasia caused by cerebrovascular accident (CVA),” Med. J. Islam Repub. Iran 32, 25 (2018). 10.14196/mjiri.32.25 30159276 PMC6108242

