# Supplementary material for: Tone disruptions in Mandarin post-stroke aphasia: an fNIRS study on Broca’s area using the auditory oddball paradigm
Source: Neurophotonics. 2025 Nov 18;13(Suppl 1):S13006. doi: 10.1117/1.NPh.13.S1.S13006 (PMC12626338; doi:10.1117/1.NPh.13.S1.S13006)
Supplement: Supplementary file 1 [file NPh_013_S13006_SD001.pdf]

Experimental Setup for Light Emission and Detection on the Scalp with MNI Coordinates of fNIRS

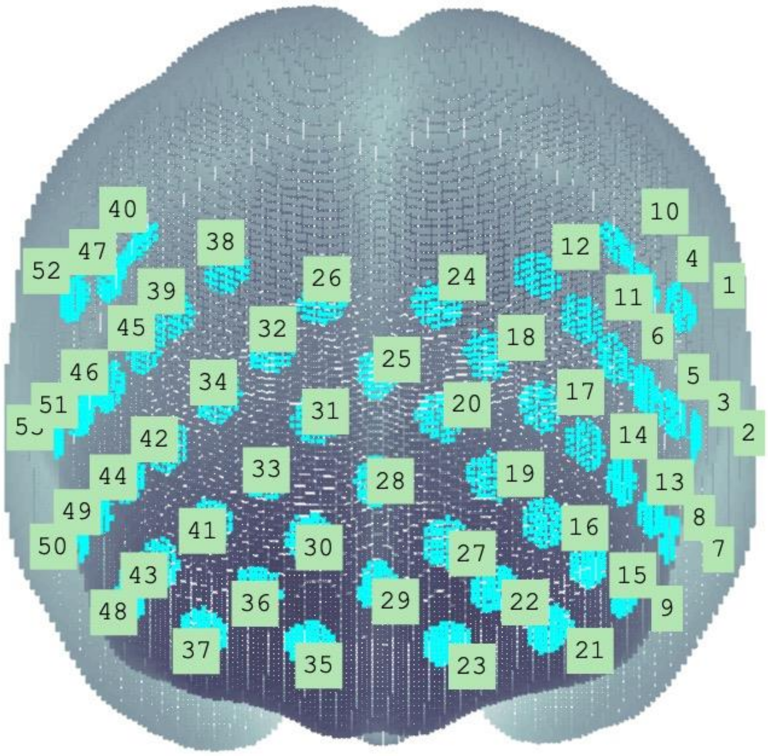

The wavelengths of the emitted light are 760 nm and 850 nm, with a frequency of 10 Hz. The distance between each emitter and detector is 3 cm. The space between the emitter and detector probes is a channel. Each probe was positioned on the forehead of the scalp. The above two images provided detail the MNI coordinates for each measurement channel.

Table: Channel Mapping to Brodmann Areas

Region of Interest (ROI)	Channels
Left Broca's Area	2nd, 3rd, 5th, 7th, 8th, 13th channels
Right Broca's Area	44th, 49th, 50th, 46th, 51st, 53rd channels
Left Dorsolateral Prefrontal Cortex (DLPFC)	6th, 11th, 14th, 17th, 18th, 20th channels
Right Dorsolateral Prefrontal Cortex (DLPFC)	31st, 32nd, 34th, 39th, 42nd, 45th channels
Left Frontal Eye Fields (FEF)	12th, 24th channels
Right Frontal Eye Fields (FEF)	26th, 38th channels
Left Frontopolar Area (FP)	9th, 15th, 16th, 19th, 21st, 22nd, 23rd, 27th channels
Right Frontopolar Area (FP)	30th, 33rd, 35th, 36th, 37th, 41st, 43rd, 48th channels
Left Motor and Supplementary Motor Cortex	1st, 4th, 10th channels
Right Motor and Supplementary Motor Cortex	40th, 47th, 52nd channels

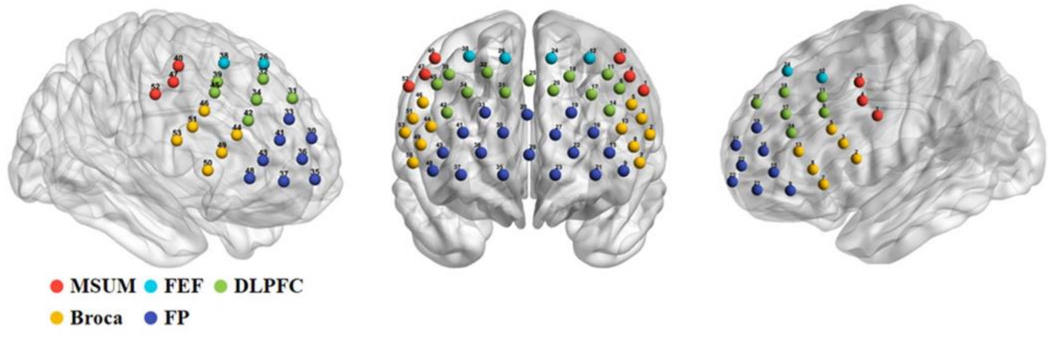

The locations of the 53 fNIRS channels are shown, with estimated cortical regions corresponding to each channel. Using the virtual registration method, these regions are categorized into the right parietal and temporal areas, the frontal area, and the left parietal and temporal areas.
